# Linking a rapid throughput plate-assay with high-sensitivity stable-isotope label LCMS quantification permits the identification and characterisation of low β-L-ODAP grass pea lines

**DOI:** 10.1186/s12870-019-2091-5

**Published:** 2019-11-12

**Authors:** Peter M. F. Emmrich, Martin Rejzek, Lionel Hill, Paul Brett, Anne Edwards, Abhimanyu Sarkar, Rob A. Field, Cathie Martin, Trevor L. Wang

**Affiliations:** 1 John Innes Centre, Norwich Research Park, Norwich, NR4 7UH UK; 2 Biosciences Eastern and Central Africa – International Livestock Research Institute, P.O. 30709, Nairobi, 00100 Kenya

**Keywords:** β-L-ODAP, BOAA, Chickpea, Grass pea, ^13^C-internal standard, *Lathyrus sativus*, LCMS, Pea, Spectrophotometric assay, Stable-isotope labelled

## Abstract

**Background:**

Grass pea (*Lathyrus sativus*) is an underutilised crop with high tolerance to drought and flooding stress and potential for maintaining food and nutritional security in the face of climate change. The presence of the neurotoxin β-L-oxalyl-2,3-diaminopropionic acid (β-L-ODAP) in tissues of the plant has limited its adoption as a staple crop. To assist in the detection of material with very low neurotoxin toxin levels, we have developed two novel methods to assay ODAP. The first, a version of a widely used spectrophotometric assay, modified for increased throughput, permits rapid screening of large populations of germplasm for low toxin lines and the second is a novel, mass spectrometric procedure to detect very small quantities of ODAP for research purposes and characterisation of new varieties.

**Results:**

A plate assay, based on an established spectrophotometric method enabling high-throughput ODAP measurements, is described. In addition, we describe a novel liquid chromatography mass spectrometry (LCMS)-based method for β-L-ODAP-quantification. This method utilises an internal standard (di-^13^C-labelled β-L-ODAP) allowing accurate quantification of β-L-ODAP in grass pea tissue samples. The synthesis of this standard is also described. The two methods are compared; the spectrophotometric assay lacked sensitivity and detected ODAP-like absorbance in chickpea and pea whereas the LCMS method did not detect any β-L-ODAP in these species. The LCMS method was also used to quantify β-L-ODAP accurately in different tissues of grass pea.

**Conclusions:**

The plate-based spectrophotometric assay allows quantification of total ODAP in large numbers of samples, but its low sensitivity and inability to differentiate α- and β-L-ODAP limit its usefulness for accurate quantification in low-ODAP samples. Coupled to the use of a stable isotope internal standard with LCMS that allows accurate quantification of β-L-ODAP in grass pea samples with high sensitivity, these methods permit the identification and characterisation of grass pea lines with a very low ODAP content. The LCMS method is offered as a new ‘gold standard’ for β-L-ODAP quantification, especially for the validation of existing and novel low- and/or zero-β-L-ODAP genotypes.

## Background

Grass pea (*Lathyrus sativus*) is a legume crop with exceptional tolerance to environmental stress factors, in particular drought and flooding [1–3]. This gives the crop considerable potential for improving food security in water-stressed areas of the world [3, 4]. Currently 25% of all agricultural land and 40% of irrigated agricultural land suffers water stress [5]. These proportions are likely to grow rapidly over the course of the twenty-first century due to the effects of climate change [6]. Despite its potential for food security and its 8000-year history of cultivation [7–10], grass pea remains greatly underutilised, primarily due to its association with the disease neurolathyrism [11–17] caused by the toxin β-*N*-oxalyl-L-α,β-diaminopropionic acid (β-L-ODAP), also known as β-*N*-oxalyl-amino-L-alanine (BOAA) or dencichine, which is produced by *Lathyrus sativus* and closely related species [18]. A few varieties with reduced β-L-ODAP contents, but no entirely β-L-ODAP-free varieties, have been released [4, 19–21]. Development of low-β-L-ODAP varieties or β-L-ODAP-free varieties has been held back by a lack of appropriate assays to screen large populations of grass pea accessions or mutants. The first assay developed to measure ODAP relied on a spectrophotometric method [22] which was later adapted by many other researchers [23, 24]. This assay is the most widely used [25–28] because of its low setup and running costs but lacks sensitivity and specificity. All variations of this assay are based on the same reaction of 2,3-diaminopropionic acid with β-mercaptoethanol and *o*-phthalaldehyde (OPA) in the presence of a tetraborate buffer system to form a yellow soluble pigment the concentration of which is measured by spectrophotometry at 420 nm. None of them, however, distinguishes between the α - and the β-isomers of ODAP and so they are subject to a slight overestimation of the toxin content since grass pea contains a small amount of the α-isomer and only the β-isomer is the active neurotoxin [29].

A variety of methods for β-L-ODAP measurement with higher sensitivity and greater accuracy than the spectrophotometric method have been described, including capillary zone electrophoresis [29], thin-layer chromatography [30] and high performance liquid chromatography (HPLC) [30–33]. Techniques using liquid chromatography mass spectrometry (LCMS) [34] or gas chromatography mass spectrometry (GCMS) [35] have been described to measure β-L-ODAP in ginseng (*Panax* spp.), but have not yet been applied to grass pea. However, these methods often rely on laborious and expensive preparation steps which limit throughput and utility in resource-poor settings. We sought to address these limitations by developing a cost-effective screening method based on parallelising the spectrophotometric method first described by Rao [22] (hereafter called the Rao assay) in a 96-well format. This simplified method is suitable for screening populations, breeding, and routine safety analyses. In addition, we describe a new, highly accurate and sensitive LCMS-based assay for measurements of β-L-ODAP content. This method, which uses a heavy-isotope-labelled internal standard of β-L-ODAP, offers a ‘gold-standard’ assay for the characterisation of future low- and zero-β-L-ODAP varieties of grass pea.

### Results

### A high-throughput spectrophotometric plate-based assay for ODAP

**Fig. 1:**
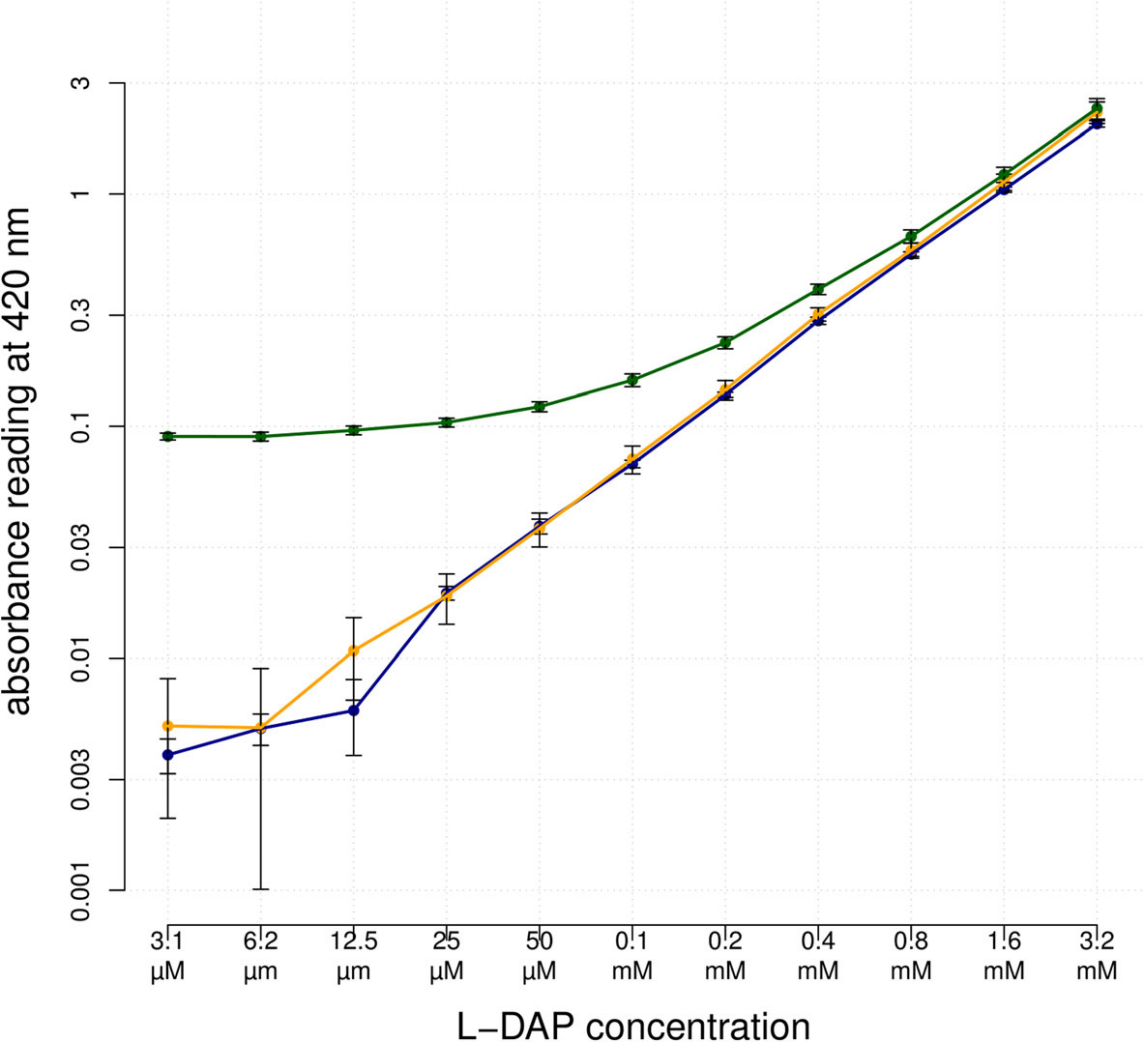
Linearity of absorbance values produced by the spectrophotometric method. Blue: serial dilution of L-DAP; green: serial dilution of L-DAP and non-hydrolysed extract without blanking; orange: serial dilution of L-DAP and non-hydrolysed extract, blanked to remove the background absorbance present in non-hydrolysed extract without L-DAP. The range of L-DAP concentrations chosen was equivalent to biologically meaningful extracts of grass pea samples of 15 mg with β-L-ODAP concentrations ranging from 0.018 to 2.25% w/w dry weight. Each datapoint represents an average of eight samples, error bars denote standard error. Both axes are logarithmic

The chemistry of the Rao assay [22] was adapted to permit the processing of many samples in parallel using a standard 96-well plate format. For screening purposes, high accuracy is not required as one wishes to reduce the noise from large populations to identify outliers (such as very low to zero-ODAP containing lines) whose ODAP content can then be measured accurately in a tiered approach using additional methods. In brief, the assay consisted of harvesting plant material directly into 96-well plates, freeze-drying the material (which can be stored at this point), pulverising and then extracting in μL volumes. For the purposes of a crude screen, pre-weighing is not necessary where the samples are of the same type e.g. young shoot tips. The extract was then sub-divided, and one part hydrolysed with KOH to generate of L-2,3-diaminopropionic acid (L-DAP) before being reacted with the Rao reagents (OPA and β-mercaptoethanol) to produce the coloured pigment. The second aliquot was reacted, in parallel, directly with the Rao reagents to provide a non-hydrolysed ‘background’ reading that was then subtracted from that of the hydrolysed extract. This removed noise due to substances other than ODAP resulting in absorbance at 420 nm [24]. The absorbances of the wells in the two plates were measured using a plate-reader spectrophotometer and the ODAP content quantified according to the calculation shown in the Methods section. Each plate contained a series of standard amounts of L-DAP for reference and ensuring consistency between plates. Figure 1 shows serial dilutions of L-DAP produced absorbance values that increased linearly with L-DAP concentration. At L-DAP concentrations lower than 0.025 mM, the absorbance measurements showed great variation between readings, indicating the detection limit of this assay. The non-hydrolysed grass pea extracts with added L-DAP produced significantly higher readings than the L-DAP standards at low L-DAP concentrations (green curve). This shows that blanking is necessary to remove the background absorbance caused by other compounds in the extract. After subtracting the absorbance measurements of the non-hydrolysed extracts alone, the readings of the blanked spiked extracts (orange curve) became indistinguishable from the readings of the dilution series for L-DAP alone. The measurement of absorbance produced by non-hydrolysed samples, therefore, is necessary to produce accurate measurements, especially at low concentrations of L-DAP/β-L-ODAP. Our plate-based assay, is sufficiently sensitive and accurate to allow the measurement of β-L-ODAP in grass pea samples and may be used to detect variation in ODAP content within large populations of material, since a single operative can process more than a thousand samples per day.

### An accurate mass spectrophotometric-based assay for β -L-ODAP

To quantify ODAP in low-ODAP samples and to demonstrate the validity of our high-throughput spectrophotometric method, we wished to develop a ‘gold standard’ method, with high specificity, sensitivity and accuracy. Such assays are frequently based on liquid chromatography and tandem mass spectrometry, using selected reaction monitoring (SRM). ODAP is a difficult analyte because it is not amenable to reverse phase chromatography; it is extremely hydrophilic and not retained. Ghosh et al. [30], recently presented an HPLC-based assay, but ODAP eluted in the dead volume of the column. With no on-column retention, ODAP cannot be selected adequately relative to other hydrophilic components in the extract. For our ‘gold-standard’ assay, we chose to derivatise ODAP to add hydrophobicity. ODAP can be derivatised by any conventional method applicable to amino acids, but we selected Waters’ AccQ-Tag™ system, based on its simplicity of use, reliability, and the high stability of the derivatised product. We chose to detect the product by tandem mass spectrometry, but AccQ-Tag™ derivatisation also offers fluorescence and UV absorbance as an alternative, albeit less accurate, means of detection. Mass spectrometry is selective and sensitive but suffers from varying efficiency of ionisation. It is also possible to lose analyte during extraction. Both sources of error can be corrected by use of an internal standard that is an isotopologue of the analyte. Since it is chemically identical to the analyte, it will suffer the same losses and the same variations in ionisation efficiency, but it can be distinguished from the analyte by the mass spectrometer, based on its increased mass.

### Synthesis of the internal standard for mass spectrometry

To produce stable-isotope-labelled β-L-ODAP we considered labelling by deuterium, ^13^C, ^15^N or combinations thereof. Based on published synthetic procedures and availability of stable-isotope-labelled starting materials we decided to ^13^C-label the oxalyl group of β-L-ODAP. An acylation of L-DAP can be achieved using methyl potassium oxalate [36] but in our hands it gave unsatisfactory results due to difficulties with preparation of the ^13^C-labelled reagent.

**Fig. 2:**
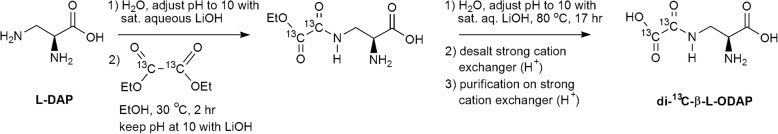
Synthesis of di-^13^C-β-L-ODAP. L-DAP was acylated in a two-stage process using diethyl oxalate-^13^C_2_ to donate the ^13^C producing the isotopologue for mass spectrometry.

**Fig. 3:**
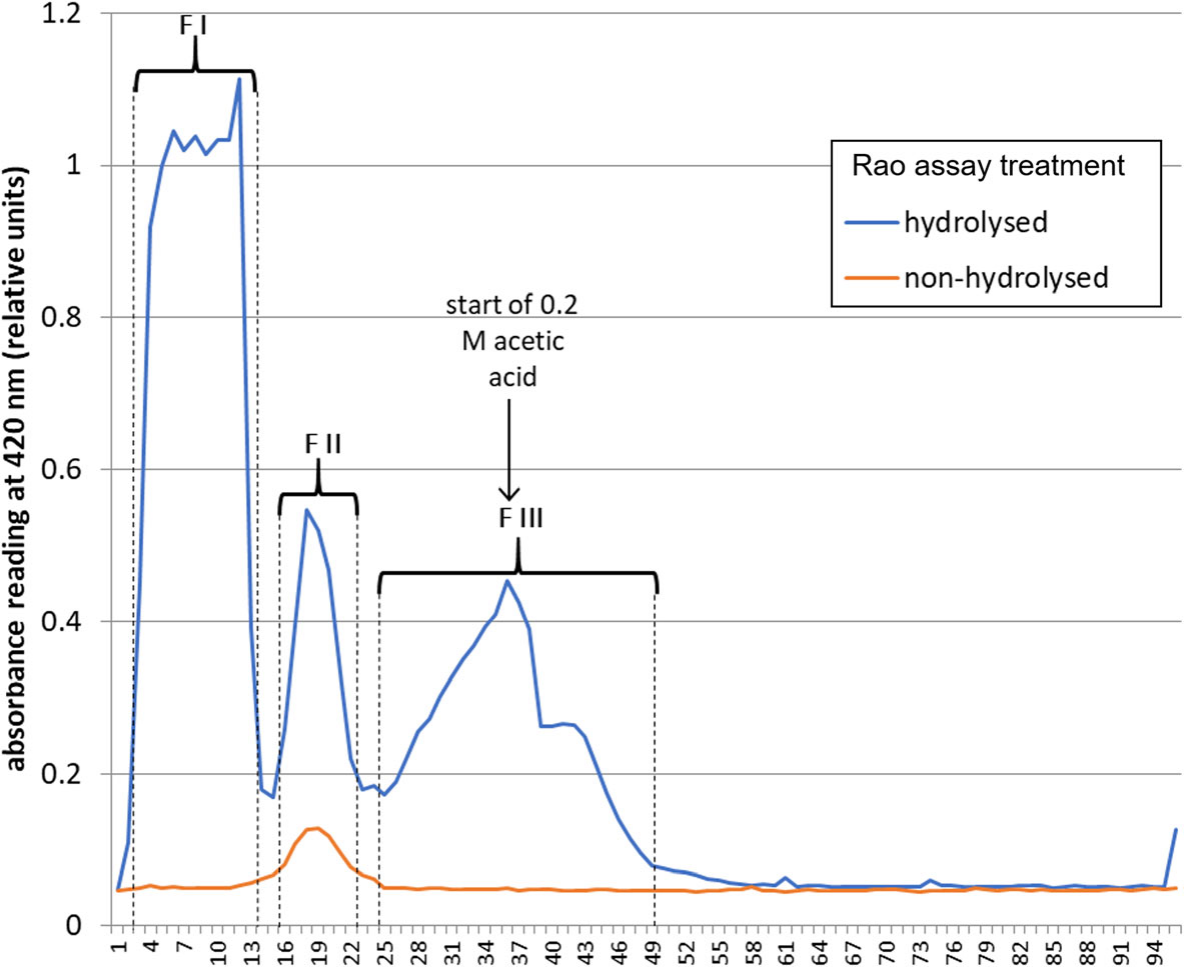
Rao spectrophotometric assay of crude reaction products of the di-13C-β-ODAP synthesis, following ion-exchange chromatography. For further analysis, vials were pooled into fractions F I, F II and F III as shown by brackets and dashed lines. No standard series was included with this experiment; hence absorbance data are shown in relative units

**Fig. 4:**
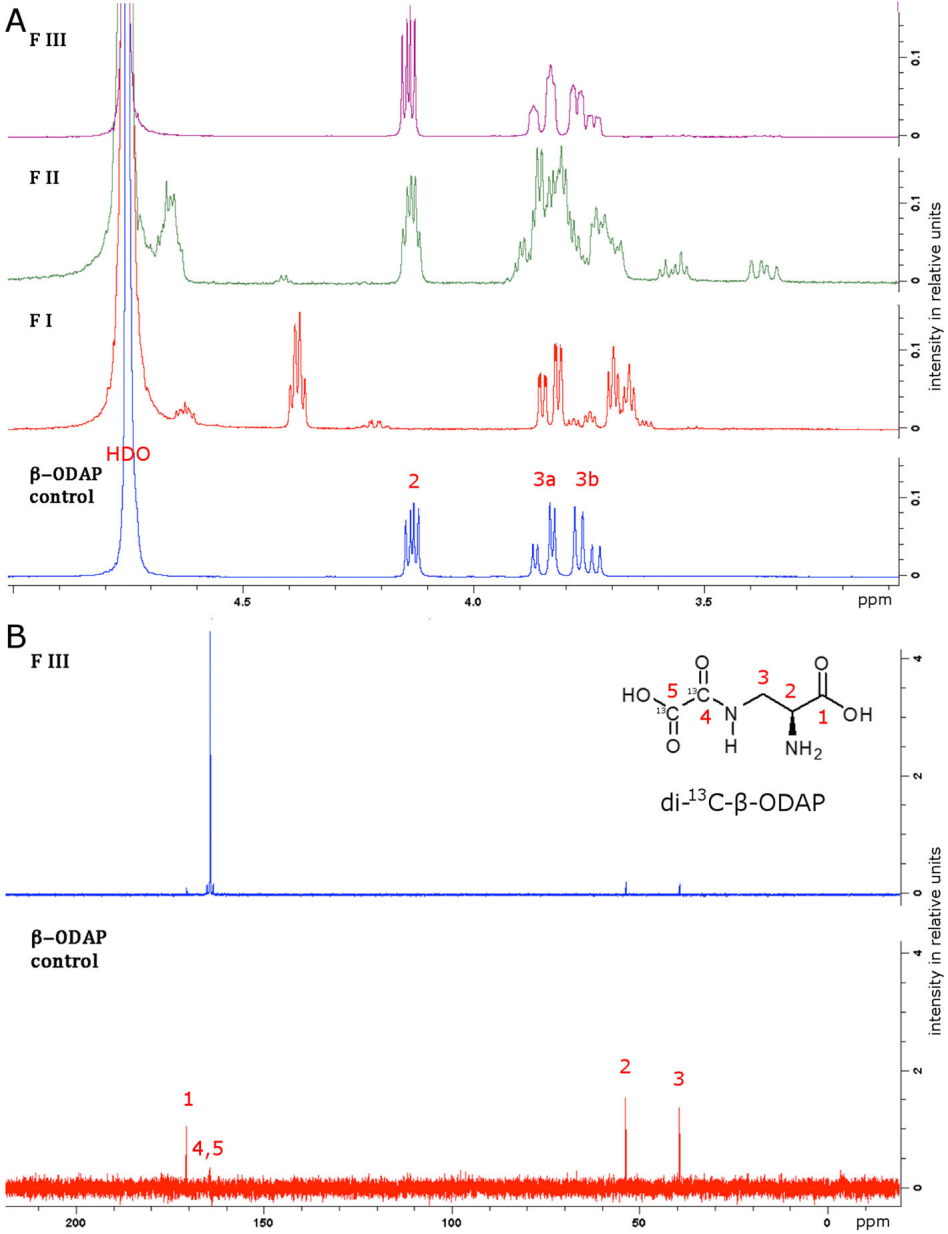
Purification of di-^13^C-β-ODAP. NMR analysis of fractions FI to FIII pooled from ion-exchange chromatography. **a** ^1^H NMR spectra of fraction FI to FIII (see Fig. 3) compared to a spectrum of an authentic β-L-ODAP standard. The spectrum of Fraction III corresponds to the β-ODAP standard. **b** ^13^C-NMR spectrum of FIII in comparison to the spectrum of authentic β-L-ODAP. Two very strong signals at 164.4 and 164.3 ppm correspond to the ^13^C-labelled oxalyl group

As an alternative, diethyl oxalate was reported [37] to bring about the acylation of the β-amino group in L-DAP (Fig. 2). When using diethyl oxalate-^13^C_2_ the reaction produced a complex mixture of intermediates that were all subjected to a base (lithium hydroxide, LiOH) catalysed ester hydrolysis. Strongly acidic cation exchange resin (Dowex 50WX8–400) was used to remove lithium cations. The desalted sample was then acidified to pH 5 with acetic acid and applied to a fresh cation exchange resin column. The column was eluted first with water followed by acetic acid. Each fraction (5 mL) was evaluated for β-L-ODAP content using the Rao assay (Fig. 3); the fractions were pooled (FI to FIII) as indicated in Fig. 3. ^1^H- and ^13^C-NMR spectroscopy were used to identify di-^13^C-β-ODAP in the three pooled fractions. The proton-NMR spectrum of Fraction III corresponded to the spectrum of an authentic β-L-ODAP standard (Fig. 4, panel a) and the spectrum was in good agreement with data reported by Abegaz et al. [38].

For final confirmation of the identity and purity of di-^13^C-β-ODAP, Fraction III was collected for ^13^C-NMR (Fig. 4, panel b). Observed peaks at 170.6, 164.4, 164.3, 53.7 and 39.4 ppm corresponded to the ^13^C-NMR spectrum acquired for the authentic β-L-ODAP standard in chemical shifts and relative intensity, except for the two peaks at 164 ppm (C4 and C5), which showed much higher intensity in the di-^13^C-β-ODAP sample, suggesting a high degree of ^13^C-incorporation in the two positions of the oxalyl moiety. No other peaks were observed, indicating the purity of the isolated di-^13^C-β-ODAP. The reaction produced a total amount of 4.2 mg of di-^13^C-β-ODAP representing 6.6% of the theoretical yield. The low yield can be explained by the production of various side products, including di-^13^C-α-ODAP, which are captured in the other fractions.

### The spectrophotometric and LCMS assays produce highly correlated estimates of β-ODAP content

**Fig. 5:**
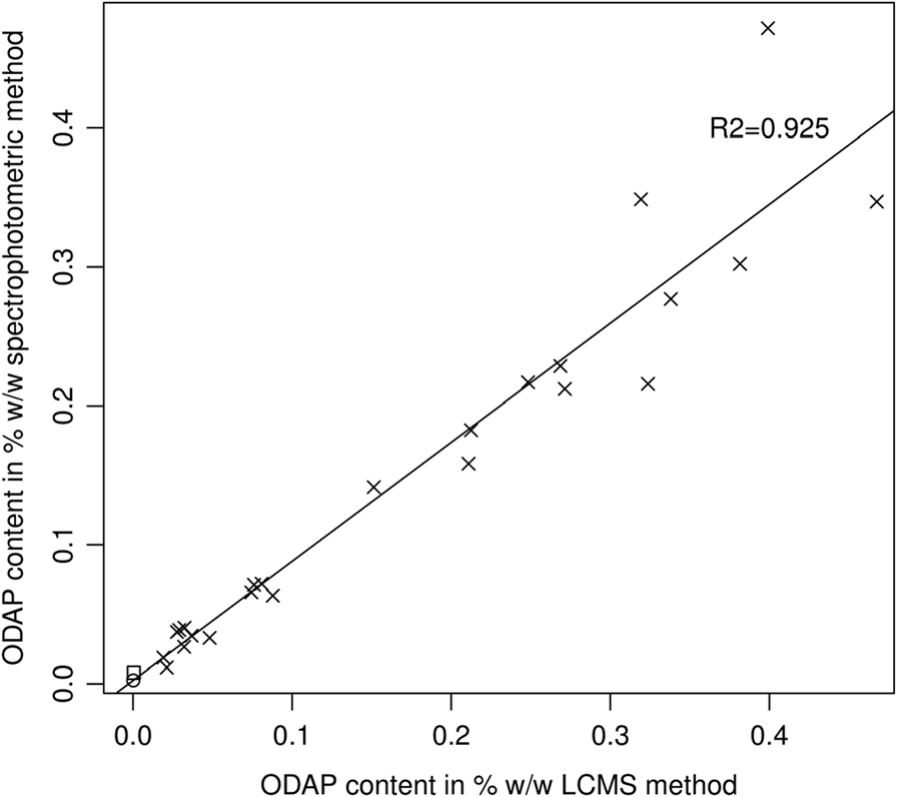
Correlation between Rao and LCMS assay measurements. ODAP measurements of grass pea seed samples (crosses), one chickpea sample (open circle) and one pea sample (open square) using the spectrophotometric and LCMS methods. A linear regression line is shown

Figure 5 shows a dataset of 24 grass pea seed samples along with one pea and one chickpea seed sample, each measured for their ODAP content using the spectrophotometric (Rao) method and our new LCMS-based method utilising the internal standard. The grass pea samples included in this study were selected to include high- and low-ODAP genotypes, with ODAP contents ranging from 0.01 to 0.47% w/w and all grown under the same conditions. The two methods were highly correlated, with a linear regression R^2^ value of 0.925. The same data are shown in Table 1, by genotype.

**Table 1:** Measured ODAP contents in % w/w in drilled individual seeds of grass pea, along with literature values. Literature values were obtained by triplicate measurements of milled bulked seed samples and thus do not reflect biological variation between individual seeds

Genotype	LCMS	std. error	Rao	std. error	Literature value	std. error	Reference
LSWT11	0.416	± 0.021	0.374	± 0.041	n/a	n/a	n/a
LS007	0.261	± 0.027	0.197	± 0.016	n/a	n/a	n/a
Nirmal	0.309	± 0.017	0.285	± 0.029	0.098	± 0.003	[39]
LS8246	0.052	± 0.016	0.036	± 0.012	0.033	± 0.004	[40]
Mahateora	0.030	± 0.001	0.039	± 0.001	0.046	± 0.001	[39]
Ceora	0.029	± 0.004	0.027	± 0.004	0.065	± 0.025	[21]
P-24	0.077	± 0.002	0.070	± 0.002	0.053	± 0.001	[39]
Pea	nil	n/a	0.008	n/a	n/a	n/a	n/a
Chickpea	nil	n/a	0.003	n/a	n/a	n/a	n/a

### Sensitive and accurate characterisation of β-L-ODAP using the LCMS assay

The two ODAP assays differed clearly in their sensitivity and selectivity for ODAP. While the methods gave good agreement for grass pea samples, for pea and chickpea samples, the spectrophotometric method produced readings equivalent to 0.008 and 0.003% ODAP respectively. In contrast the LCMS method found no detectable β-L-ODAP in either sample (< 0.0005%; Table 1).

The specificity of our LCMS-based method is based on the retention time during chromatography, the mass of the precursor ion, and the fragment formed from the precursor, making this method complementary to the Rao assay. In the case of AccQ-Tag™-derived amino acids, the predominant fragment is the tag itself. Thus, the mass transitions we used to quantify β-L-ODAP and di-^13^C-β-ODAP were *m/z* 347.1 → 171.1 and 349.1 → 171.1, respectively. The method is specific for amine compounds with the appropriate molecular weight and retention time; chickpea and pea contain no such compounds.

**Fig. 6:**
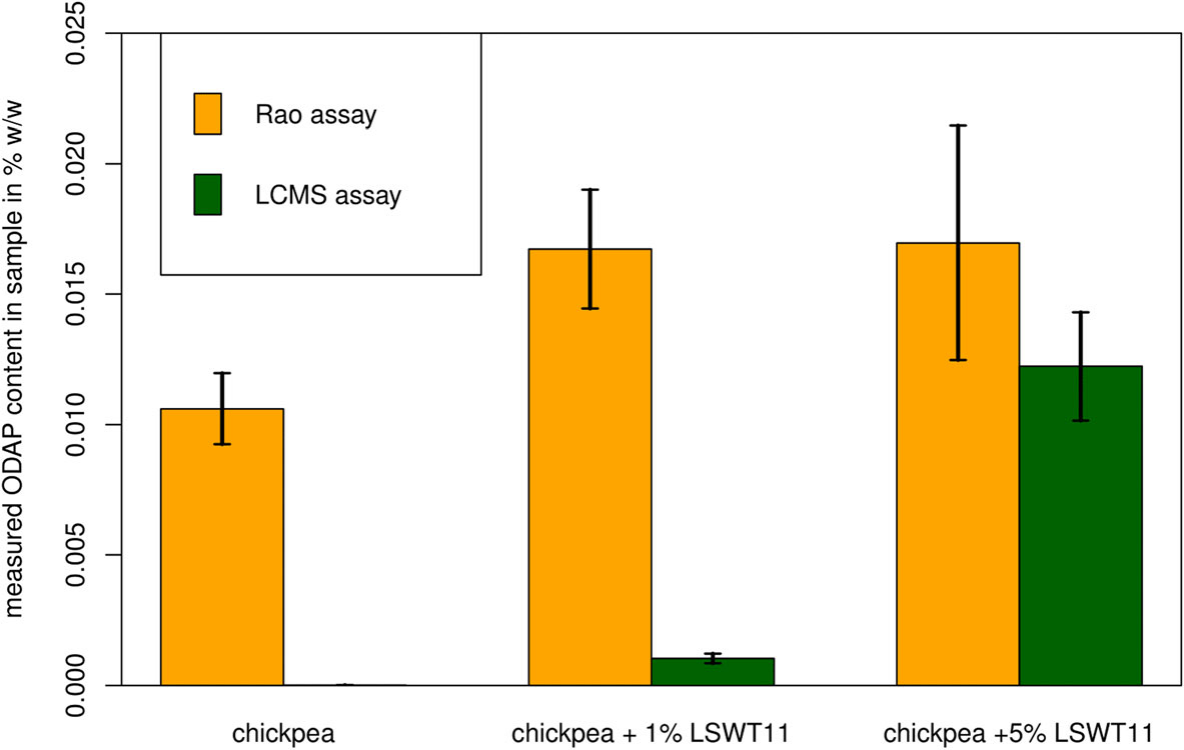
ODAP measurements in chickpea seed meal samples spiked with grass pea seed meal. Averages of three extractions are shown, error bars denote standard errors

The sensitivity of any assay is limited by its selectivity, in that it is impossible to detect or quantify a low concentration of the correct analyte in the presence of a high level of background signal indistinguishable from analyte. To test the sensitivity of the LCMS and spectrophotometric assays, we measured ODAP content in samples of chickpea seed meal spiked with 1% or 5% of grass pea seed meal (LSWT11), with results shown in Fig. 6. The average recovery of di-^13^C-β-ODAP across all seed meal samples was 80.0% ± 6.6% std. error. Average recovery of di-^13^C-β-ODAP across the standard series was 100.0% ± 7.5% std. error. The spectrophotometric assay did not show a significant difference between the 1 and 5% grass pea samples, indicating that at this very low abundance, corresponding to 0.01% w/w ODAP, the spectrophotometric assay is limited to detection of the presence of ODAP but cannot reliably quantify the amount present. The LCMS-based assay allowed quantification of β-L-ODAP in the 1% spiked samples, corresponding to a β-L-ODAP concentration of 0.002% w/w. No peak corresponding to β-L-ODAP was detected in the pure chickpea sample using the LCMS-assay.

**Fig. 7:**
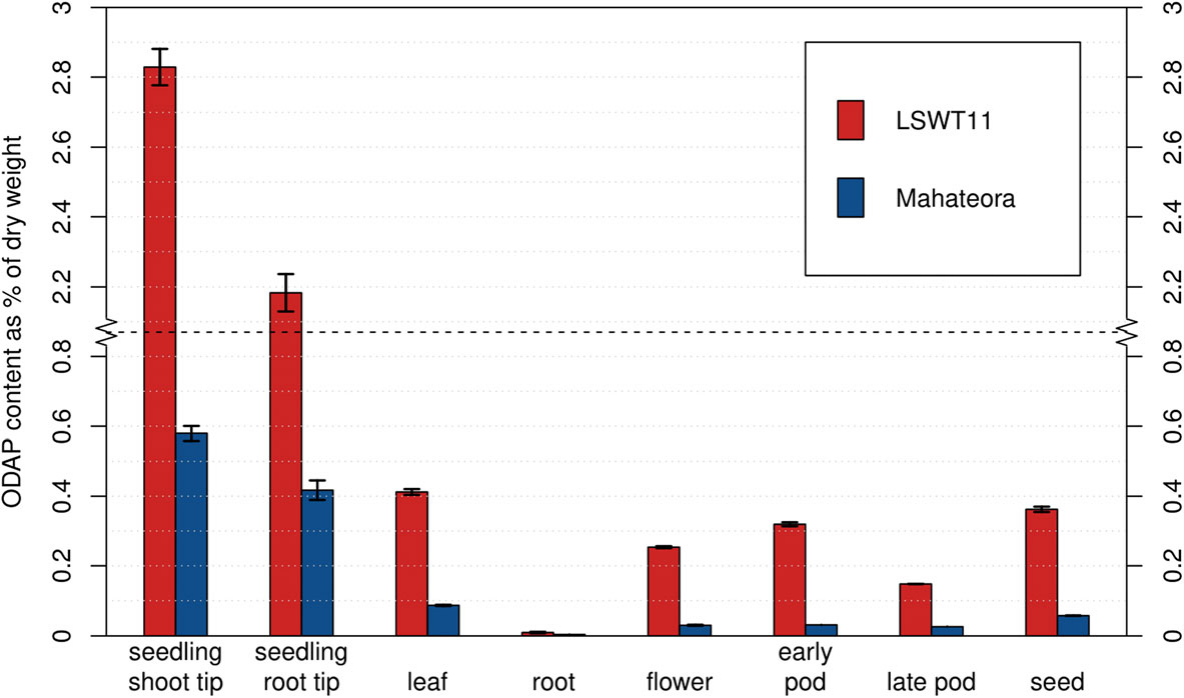
β-L-ODAP content in eight tissues of the Indian high-ODAP grass pea landrace LSWT11 and the low-ODAP variety Mahateora, measured by LCMS

Using the LCMS-assay with the internal standard prior to extraction, we quantified β-L-ODAP contents in multiple tissues of the Indian high-ODAP landrace LSWT11 and the low-ODAP variety Mahateora (Fig. 7). The eight tissues tested were seedling shoot tip, seedling root tip, leaves, roots, flowers, early pods, late pods and seeds. This revealed large variations in β-L-ODAP content between different tissues, in the case of LSWT11 ranging from 2.829% ± 0.052% (std. error) of dry weight in seedling shoot tips to 0.010% ± 0.002% in roots, with 0.362% ± 0.007% in seeds. Measurable concentrations of β-L-ODAP were found in all tissues, though only trace amounts were present in mature roots.

In each tissue, the β-L-ODAP content of Mahateora was significantly lower than LSWT11 at the *p* < 0.001 level, with the exception of roots where the difference was not significant (*p* > 0.08). Nevertheless, β-L-ODAP was detected in all tissues of Mahateora, ranging from 0.579% ± 0.021% w/w in seedling shoot tips to 0.004 ± 0.000% in roots, with a seed β-L-ODAP content of 0.058% ± 0.001%.

## Discussion

The determination of ODAP in plant tissues has long-relied on the established spectrophotometric method of Rao [22]. This uses the difference of absorbance measurements at 420 nm between hydrolysed and non-hydrolysed samples following treatment with *o*-phthalaldehyde. Its specificity is based on both the hydrolysis and the colour-forming reaction. In most applications, *o*-phthalaldehyde reacts with amino acids to yield fluorescent products without absorbance at 420 nm, ODAP being an exception. Rao assigned the behaviour of ODAP to its vicinal amine groups [22]. The Rao assay is thus a measure of any compound that hydrolyses yielding vicinal amine groups, and it is possible that the false positive measurement from pea and chickpea indicates the presence of another such compound, at low levels. If any such compounds also exist in grass pea, it may result in an overestimation of the true ODAP concentration. The limitations of the Rao method become apparent when seeking grass pea lines that contain low amounts of β-L-ODAP. First, is the inability of the spectrophotometric method to differentiate between β-L-ODAP and its non-toxic isomer α-L-ODAP [41], which represents approximately 5% of total ODAP in grass pea tissues. This is because the method relies on the alkaline hydrolysis of ODAP to L-DAP, and the same compound is produced by the hydrolysis of both isomers of ODAP [38]. The measurements produced by the spectrophotometric method are, therefore, a composite measurement of total ODAP, rather than of β-L-ODAP alone. Second, as mentioned above, the spectrophotometric assay appears to detect compounds other than ODAP. It therefore tends to overestimate the active ODAP content. The issues of technical variation and background are also particularly relevant when comparing ODAP contents in different tissues, as the background varies between tissues caused by other compounds absorbing light at 420 nm wavelength in the spectrophotometric assay. When measuring green tissue samples of low-ODAP genotypes, this can lead to negative ODAP values as the noise caused by sample variation swamps the signal generated by ODAP. This makes the spectrophotometric method unsuitable for measuring ODAP contents in low-ODAP, green tissues. However, by developing the Rao-based high-throughput spectrophotometric method outlined here and linking it to a more accurate LCMS method, it becomes possible to identify low or no β-L-ODAP-containing grass pea lines. One final consideration when measuring ODAP is that content is heavily influenced by environmental variations, including year-to-year variations in the same field [31, 40]. To compare the ODAP contents of different genotypes, therefore, it is necessary to grow the tissues used for the experiment under consistent conditions. The assays described here allow the genetics underlying this environmental response to be studied if coupled with a population genomic dataset for a metabolic whole genome association study (mGWAS) or genotyping data segregating population to identify metabolic quantitative trait loci (mQTL). Understanding the effects of Genotype x Environment interactions on β-L-ODAP is crucial to direct future breeding efforts. The synthesis and use of a stable, heavy-isotope-labelled version of the β-L-ODAP molecule enables the determination of the natural compound with greater accuracy than in previous studies. Techniques involving stable-isotope-labelled (SIL) internal standards are considered the ‘gold standard’ in LCMS analyses [42]. If the SIL standard is included in the sample prior to extraction or any processing, it behaves like the chemically near-identical natural compound. However, due to its different molecular weight, it can be distinguished in the mass spectra, providing an intensity peak corresponding to a known quantity of standard, thus accounting for any losses during extraction and processing. The synthesis we describe here produced a heavy-isotope-labelled standard of high purity, although the yield of this reaction was low (6.6%). Using the relatively inexpensive substrate L-2,3-diaminopropionic acid in excess would result in a greater proportion of the unwanted dipeptide side product being formed. However, because only very small amounts of the internal standard are needed in the assay, a single synthesis was sufficient to test several hundred samples.

The internal standard also provided a means to confirm the absence of β-L-ODAP from a sample, because it excludes the possibility that a small amount of β-L-ODAP in the sample is masked by a chemical effect that impacts elution on the HPLC column or ionisation in the mass spectrometer – such effects would also affect the SIL-standard [42–44]. Hence, the LCMS method using the internal standard could be used to characterise any future zero-ODAP grass pea genotypes. The cost and complexity of synthesising the SIL standard may make this approach unsuitable for the testing of large collections of samples, but it provides a ‘gold standard’ method to characterise new genotypes.

The assay described here offers an extremely wide dynamic range. The standard series ranges from ODAP concentrations equivalent to 1% down to 0.00046% dry weight. The β-L-ODAP contents determined in different tissues of LSWT11 and Mahateora were spread across about a 1000-fold range (between LSWT11 seedling shoot tip and Mahateora roots) but were quantified in a single experiment. Due to the high sensitivity of the triple-quad LCMS system, this range could be extended by using less or more than the 500-fold dilution following derivatisation. The new LCMS-based method showed a high degree of agreement (R^2^ = 0.925) with the plate-based version of the widely used spectrophotometric ODAP assay. While less sensitive than the LCMS-based assay and susceptible to interference from other compounds absorbing at the wavelength of 420 nm, the spectrophotometric assay is a cheap method of screening hundreds of samples in one experiment, e.g. for the use in mutant screens, and routine safety controls of field grown material. However, for accurate determination of β-ODAP concentrations in potentially low-ODAP varieties, the LCMS assay is superior.

To extract β-ODAP from tissue samples, methods using deionised water at 100 °C [24], 60% ethanol at room temperature [22, 23], and 70% at room temperature [45] have been described. Several extraction media were compared by Hussain et al. [24], who found no significant differences in β-ODAP measurements. Which extraction medium is most suitable also depends on the tissue used. When extracting seed meal with water at 95 °C, the high carbohydrate content causes a heterogeneous gel to form. Conversely, extraction with ethanol also extracts chlorophyll from green tissues, increasing noise in the spectrophotometric assay. This is not an issue for the LCMS assay which includes extraction with ethanol, evaporation and re-dissolving in water and does not rely on absorbance. Applying the LCMS method to compare a low- and high-ODAP containing grass pea varieties, the seed β-L-ODAP content measured for the variety Mahateora (0.06% of dry weight) was below the commonly applied threshold [46, 47] for low-ODAP genotypes of 0.1%, and below results obtained by others working with this genotype [48]. The quantification of β-L-ODAP in eight tissues of the genotypes LSWT11 and Mahateora revealed that the low-ODAP phenotype was observed in all these tissues, with β-L-ODAP contents between 2.5 times (roots) and 10.5 times (early pods) lower in Mahateora than in LSWT11. These data indicate that whatever factor(s) cause the low-ODAP phenotype in Mahateora, they do not affect the distribution of the toxin in the plant but involve a global reduction on β-L-ODAP biosynthesis or a change in β-L-ODAP breakdown.

As mentioned, our LCMS method resolves the two ODAP isomers by liquid chromatography, with β-L-ODAP eluting slightly earlier than α-L-ODAP, allowing both compounds to be measured. Equally, the LCMS assay allows direct measurement of L-DAP, the proposed final intermediate of β-L-ODAP synthesis. L-DAP cannot be directly determined using the spectrophotometric method, because the absorbance produced by any L-DAP already present in the sample would contribute to the absorbance of both the hydrolysed and non-hydrolysed samples and could not be distinguished from the background absorbance caused by other sources. The LCMS method described in this study, therefore, represents the first highly sensitive method capable of measuring β-L-ODAP, α-L-ODAP and L-DAP simultaneously. Despite a low threshold of detection, quantifiable levels of L-DAP were not observed in any grass pea tissue.

## Conclusions

The plate-based spectrophotometric assay described here allowed fast and cost-efficient quantification of ODAP in large collections of grass pea samples for the purposes of germplasm screening and breeding. It can be adapted further to screen very large populations qualitatively, e.g. for the identification of low- or high-ODAP lines from mutagenised populations. The high-sensitivity LCMS assay using a heavy-isotope-labelled internal standard represents a new ‘gold-standard’ assay for β-L-ODAP that can be used to characterise novel grass pea genotypes. Due to its low threshold of detection, this method would be the assay of choice to characterise any future zero-β-L-ODAP genotypes, once this critical limitation (β-L-ODAP content of seed) preventing the widespread use of grass pea for food security has finally been removed.

## Methods

### General

L-2,3-diaminopropionic acid hydrochloride and diethyl oxalate-^13^C_2_ were purchased from Sigma-Aldrich. NMR spectra were recorded on a Bruker Avance III 400 MHz spectrometer. Chemical shifts of ^1^H NMR signals recorded in D_2_O are reported with respect to residual HDO at δ_H_ 4.75 ppm.

Seeds of the variety Ceora were supplied by Prof Kadambot Siddique, University of Western Australia, Perth, Australia. LS8246 seeds were obtained from the USDA germplasm collection, Pullman, WA, USA. Pea seeds (variety Cameor) and seeds of LSWT11, LS007 and the varieties Nirmal, Mahateora and P-24 were obtained from the John Innes Centre germplasm collection in Norwich, UK. LSWT11 and LS007 have been deposited with NBPGR India under accession numbers EC859045 and EC862759, respectively. All seed materials used in this study were multiplied in glasshouses at the John Innes Centre. Chickpea seed meal (KTC Gram flour) was obtained commercially (product number 7565125, J Sainsbury plc, London, UK).

### Preparation of plant material

Shoot tips. Young shoot tip tissues (5–10 mm in length) were taken from seedlings 7–9 days after sowing in compost (12 × 19-well trays of John Innes No. 2 compost with 20% grit) and growing in a glasshouse (minimum 15 °C, unlit). To enhance simultaneous germination, seeds were scarified by rubbing for ca. 15 s on 120-grit aluminium oxide sandpaper before sowing. Shoot tips were collected in 96-well microtitre plates on dry ice and freeze-dried for 36 h. To the dry material in each well a 4 mm steel bead was added and the sample pulverized by shaking for 1 min at 18 Hz.

Seeds. Three individual mature dry seeds of eight genotypes of grass pea, the original Indian “low-ODAP” variety P-24 [47] (now classified as intermediate type), the low-ODAP Indian varieties Mahateora [45] and Ratan [20] derived from P-24, the intermediate-ODAP Indian variety Nirmal [48], the high-ODAP line LSWT11, the low-ODAP variety Ceora [21] from Australia, the low-ODAP genotype LS8246 [40] from Canada and the high-ODAP European genotype LS007 from the UK. All seeds used in this experiment were collected from plants grown in the glasshouse at the JIC.

Sample material was obtained from seeds by drilling through cotyledons (benchtop drill, Xenox, Föhren, Germany) to extract seed meal. Seeds of pea and chickpea were also included as controls since they are expected to contain no ODAP. Meals were freeze-dried and accurately weighed samples (ca. 25 mg) were taken for calculation of β-ODAP concentrations.

Multi-tissue characterisations. Seedling shoot tips and seedling root tips were collected from 7-day-old seedlings germinated in sterile water. Leaves and roots were harvested from 5-week-old plants grown in flasks containing a sand, perlite, vermiculite 2:1:1 volume mixture. The below-ground section of each flask was covered in black plastic. Flowers, early pods and late pods were harvested from 2-month-old plants grown in soil a glasshouse. Mature seeds were harvested from the same plants, 4 months after planting. Three biological replicates were prepared for each tissue. Individual seeds were drilled as above. All other tissues (seedling shoot tip, seedling root tip, leaves, roots, flowers, early pods and late pods) were collected in liquid nitrogen and ground using a pestle and mortar chilled with liquid nitrogen. The sample powders were freeze-dried overnight and approximately 6.5 mg of dry powder of each sample was weighed out, recording accurate weights for calculating ODAP content.

### Parallelised 96-well plate modified Rao assay

This method is based on the spectrophotometric method developed by Rao et al. [22] and adapted by Briggs et al. [23, 24]. This method was further modified by scaling down reagent quantities and adjusting operational steps to allow parallelised processing of samples in 96-well microtitre plates.

ODAP was extracted from the samples by adding 600 μL of 60% v/v ethanol in deionised water to each ground sample and incubating at room temperature overnight in a shaking incubator to keep the samples in suspension. After centrifugation of the plates for 10 min at 2500 x g, ODAP content was measured by withdrawing an 80 μL aliquot into a 96-well microtitre plate (Sterilin) containing 160 μL of 3 M KOH (potassium hydroxide) solution. Plates were sealed firmly and clamped tightly to prevent leakage. The plates were submerged in a water bath at 95 °C for 30 min to hydrolyse ODAP to L-DAP. After this, the plates were submerged in water at room temperature to cool them down before drying and releasing the clamps, to prevent the ethanol in the solution from boiling off.

The *o*-phthalaldehyde/tetraborate reaction buffer was prepared by dissolving 5.29 g of potassium tetraborate tetrahydrate (Sigma-Aldrich) in 42 mL of dH_2_O, by shaking and gently warming the mixture. Separately, 34.6 mg of *o*-phthalaldehyde (Sigma-Aldrich) were dissolved in 346 μL of absolute ethanol and 69 μL of β-mercaptoethanol (Sigma-Aldrich). Once both *o*-phthalaldehyde and potassium tetraborate were fully dissolved, the solutions were combined. The resulting 42.4 mL of reagent buffer were sufficient for processing 96 samples in parallel in two microtitre plates, one each for hydrolysed and non-hydrolysed aliquots. For the colour-forming reaction, 30 μL of hydrolysate was mixed with 220 μL of OPA/tetraborate buffer in a 96-well microtitre plate with a clear flat bottom (Greiner Bio-One).

Simultaneously, another flat-bottom plate was loaded with 20 μL of 3 M potassium hydroxide solution and 10 μL of non-hydrolysed supernatant from the overnight extraction, immediately followed by 220 μL of OPA/tetraborate buffer. At room temperature and without allowing the incubation time, the hydrolysis reaction does not occur. However, it is nevertheless necessary to add KOH to the non-hydrolysed treatment, as its presence affects absorbance at 420 nm.

For accurate measurement of ODAP in a smaller number of samples, for example, in germplasm screening, known weights of material were used. Free amino acids, including L-2,3-diaminopropionic acid (L-DAP) and β-L-ODAP, were extracted from the seed meal in 600 μL of 60% ethanol in distilled water (v/v) and incubated at room temperature with shaking for 22 h. Samples were centrifuged at 16,250 x g in a benchtop centrifuge for 10 min. A series of standards was included with each plate of samples for accurate quantification of ODAP, comprising one blank and 11 L-DAP.HCl concentrations ranging from 0.0044 mg/mL to 0.176 mg/mL, dissolved in deionised water. An aliquot (10 μL) of each standard solution was mixed with 20 μL of 3 M KOH solution and 220 μL of OPA reagent buffer. L-DAP does not require hydrolysis to react with OPA and β-mercaptoethanol in the colour forming reaction, but the addition of KOH is necessary to adjust the pH to be the same in the samples.

The colour-forming reaction mixture in each well was mixed by gentle sideways tapping the plate and left to incubate at room temperature for 30 min. Absorbance at 420 nm was measured using an optical plate reader (VersaMax, Molecular Devices). For each sample, the colour-forming reaction was carried out with and without first undergoing hydrolysis treatment at 95 °C. The absorbance measurement of the non-hydrolysed sample was subtracted from the absorbance measurement of the hydrolysed sample to exclude any absorbance caused by compounds other than ODAP. The slope of the linear regression was used to calculate seed ODAP concentrations using the following formula taking into account the dilutions used during extraction:

$$ conc=\frac{\left({A}_{hyd}-{A}_{non- hyd}\right)\times {V}_{ext}}{m_{sample}\times {a}_{standard}}\times 100\% $$

**Table:** 

conc	*seed ODAP concentration in % w/w*
*A*_*hyd*_	*absorbance reading of hydrolysed sample after the colour- forming reaction (averaged across technical replicates) in relative absorbance units (RAU)*
*A*_*non-hyd*_	*absorbance reading of non-hydrolysed sample in RAU*
*V*_*ext*_	*Volume of extraction buffer in mL*
*m*_*sample*_	*Mass of the seed meal sample in mg*
*a*_*standard*_	Slope of the standard curve in RAU*mL/mg

### Synthesis of di-^13^C-β-ODAP

The heavy-isotope-labelled internal standard was synthesised using the method of Harrison et al. [37]. L-2,3-Diaminopropionic acid hydrochloride (50 mg, 0.36 mmol) was dissolved in deionised water (0.9 mL). The pH of the solution was adjusted to 10 using LiOH (saturated aqueous). To this solution, diethyl oxalate-^13^C_2_ (0.5 mL, 3.7 mmol) dissolved in ethanol (0.54 mL) was added dropwise over the course of 2 h, while stirring at 30 °C and maintaining the solution at pH 10 by addition of LiOH. The reaction mixture was stirred for an additional 30 min and then evaporated to dryness under vacuum. The residue was dissolved in water (25 mL) and adjusted to pH 10 using LiOH. The stirred mixture was hydrolysed by heating to 70 °C for 20 h.

After cooling to room temperature, the reaction mixture was passed through a column of pre-washed ion-exchanger (10 g, Dowex 50WX8–400 in H^+^ form) to remove Li^+^ ions. The column was eluted with water and the eluate (100 mL) was freeze dried. The desalted sample was dissolved in water (50 mL) and adjusted to pH 5 using glacial AcOH. The solution was applied to a column of pre-washed ion-exchanger (19.8 g, Dowex 50WX8–400 in H^+^ form) and eluted with water (100 mL) followed by aqueous AcOH (0.2 M, 300 mL). Fractions (5 mL) were collected and an aliquot from each fraction was examined by the Rao assay. Fractions containing the product were pooled and freeze dried to give pure di-^13^C-β-ODAP (4.2 mg, 6.6%). NMR spectra are shown in Fig. 4. ^1^H NMR (D_2_O, 400 MHz): *δ*_H_ 4.14 (1H, dd, ^*3*^*J*_2,3a_ = 4.0 Hz, ^*3*^*J*_2,3b_ = 7.0 Hz, H2), 3.87–3.82 (1H, m, H3a), 3.78–3.72 (1H, m, H3b). ^13^C NMR (D_2_O, 100 MHz): *δ*_C_ 170.6 (1C, C1), 164.4 and 164.3 (2C, C4 and C5), 53.7 (1C, C2), 39.4 (1C, C3). The ^1^H NMR spectrum was in good agreement with the literature [38, 50].

### LCMS quantification of β-L-ODAP

The ^13^C-labelled β-L-ODAP standard was dissolved in deionised water to make a 250 μg/mL solution. 10 μL of this internal standard solution was added to each sample prior to extraction. To quantify accurately the tissues with expected ODAP contents above 1% of dry weight (LSWT11 seedling shoot tip and seedling root tip), 40 μL of internal standard were added. After extraction, these samples were diluted 4-fold to bring their ODAP concentrations to within the range of the standard curve.

Extractions were performed using the method described by Kuo et al. [49] for the extraction of free amino acids, including β-L-ODAP, from Asian ginseng (*Panax ginseng*). To each sample of finely ground, freeze-dried tissue, 500 μL of 70% HPLC-grade ethanol in RO-water were added and left to extract overnight in a shaking incubator. Samples were centrifuged for 30 min at 16,250 x g. Supernatants were aspirated into new tubes and pellets were re-suspended in 500 μL of 70% ethanol and centrifuged twice more. The supernatants of the three extraction cycles were combined, dried in a EZ-2 evaporator (GeneVac, Ipswich, UK) and re-dissolved in 1 mL of RO-water.

A series of β-L-ODAP (Lathyrus Technologies, Hyderabad, India) standards was prepared by serial dilution of a 0.05 mg/mL solution. This resulted in a range of standard solutions equivalent to extracts from 5 mg grass pea tissue samples with β-L-ODAP contents ranging from 0.00046 to 1% dry weight. The heavy-isotope-labelled standard was added to each of these standard solutions to a concentration of 2.5 μg/mL, the same concentration as in the sample extracts.

**Fig. 8:**
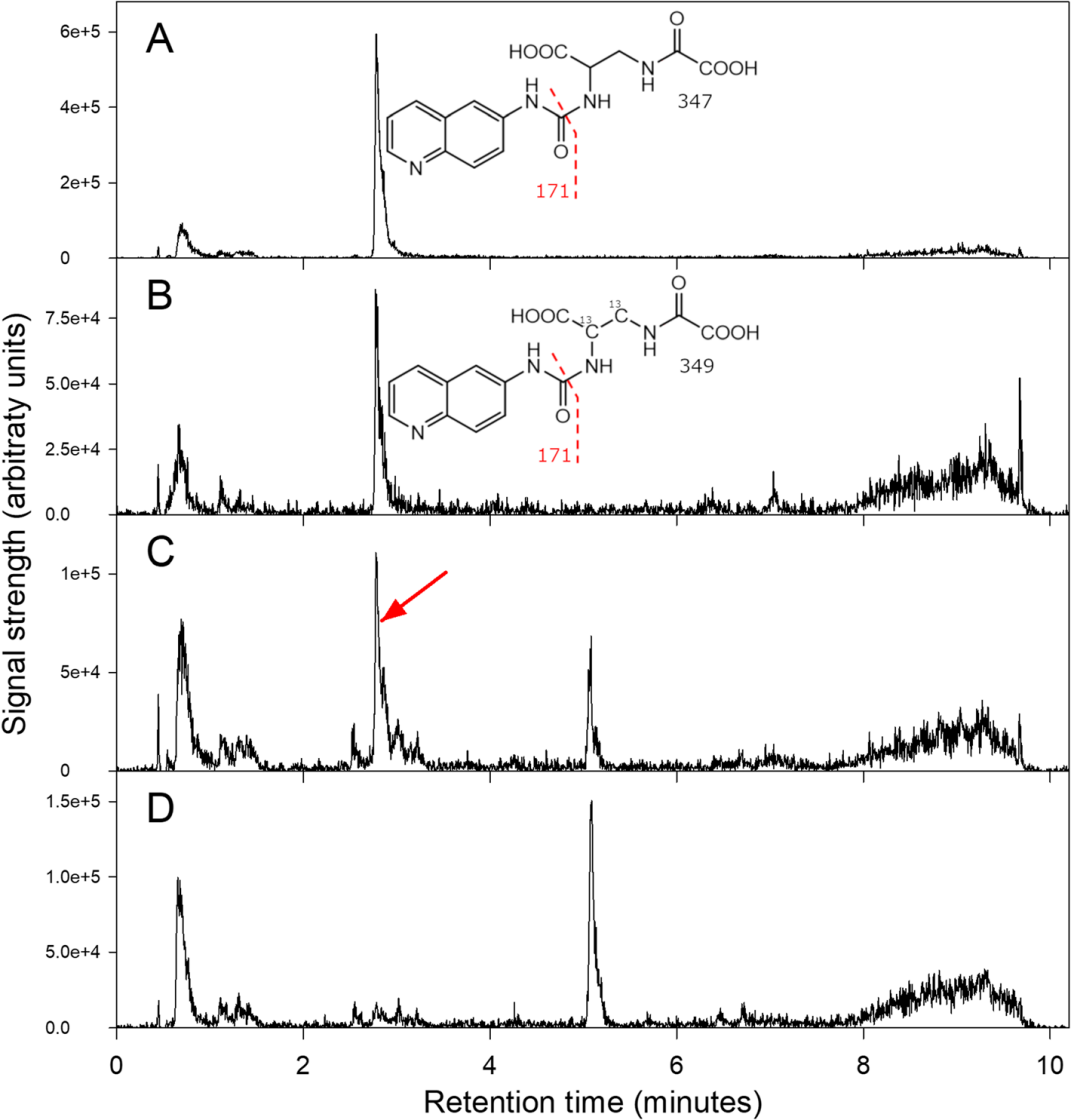
Chromatograms of AccQ-Tag™-derivatised plant material and standards. **a**: Mass transition 347 → 171 for derivatised ^12^C-ODAP standard. **b**: Mass transition 349 → 171, for derivatised di-^13^C-ODAP internal standard, in a sample of chickpea seed meal with a 1% admixture of LSWT11 grass pea meal; note that the internal standard peak is clearly-defined, with no nearby interfering peaks. **c**: Mass transition 347 → 171 in the same sample as (B); note the clearly defined peak corresponding to derivatised ^12^C-ODAP derived from the sample. **d**: Mass transition 347 → 171 in a sample of chickpea seed meal without admixture; note that the ODAP peak is now missing. Taken together, traces (C) and (D) indicate the clarity and specificity with which the LC-MS-based assay can detect ODAP at a level corresponding to 0.004% in a biological background of seed meal

Sample extracts and β-L-ODAP standards were derivatised using AccQ-Tag™ reagent (Waters, Milford, Massachusetts, USA) following the manufacturer’s instructions. Briefly, 20 μL of each sample extract and each standard solution were mixed with 60 μL of AccQ-Tag™ borate buffer. To this, 20 μL of dissolved AccQ-Tag™ reagent were added, mixed immediately and incubated at 55 °C for 10 min. The derivatised samples were diluted 1:500 (in three dilution steps) before LCMS analysis. Chromatograms of AccQ-Tag™-derivatised standards and plant tissue samples are shown in Fig. 8.

Derivatised samples and standards were quantified using a Xevo triple quadrupole TQ-S instrument (Waters, Milford, Massachusetts, USA). Separation was on a 100 × 2.1 mm 2.6 μm 100 Å Kinetex EVO C18 column (Phenomenex) using the following gradient of acetonitrile (solvent B) versus 0.1% formic acid in water, run at 0.4 mL.min^− 1^ and 30 °C: 0 min, 1% B; 0.4 min, 1% B; 7 min, 25% B; 8.5 min, 90% B; 9.0 min, 90% B; 9.1 min, 1% B; 12.6 min, 1% B. Detection was by positive mode electrospray MS using the following mass transitions, each compound being detected in its AccQ-Tag™-derivatised form: for β-L-ODAP, 347.1 → 171.1; for di-^13^C-β-L-ODAP, 349.1 → 171.1; for L-DAP, 275.1 → 171.1 and 445.1 → 171.1. The cone voltage in each case was 4 V, and the collision energy 18 V. These conditions were optimised using the automated method development tool (Intellistart) provided with Waters’ MassLynx software; the test standards were derivatised β-L-ODAP and L-DAP. Spray chamber conditions were 600 °C desolvation temperature, 1000 L.hr.^− 1^ desolvation gas, 150 L.hr.^− 1^ cone gas and 6.0 bar nebuliser pressure. Quantification was carried out using TargetLynx software.

To assess the strength of the correlation between the results of the spectrophotometric and LCMS-based methods, a linear regression model was generated using the R version 3.6 lm() function.

### NMR

Proton and ^13^C spectra were measured using a Bruker Avance III 400 MHz NMR instrument (Bruker, Billerica, Massachusetts, USA). Samples were dissolved in 500 μL D_2_O in 5 mm NMR tubes. Analysis of spectra was performed using the TopSpin version 3.2 software package (Bruker, Billerica, Massachusetts, USA).

## Data Availability

All plant materials used in this article are available from the corresponding author. The datasets used and analysed during the current study are available from the corresponding author on reasonable request.

## Abbreviations

BOAA: β-N-oxalyl-amino-L-alanine (synonym for β-L-ODAP)
GCMS: Gas chromatography mass spectrometry
HPLC: High performance liquid chromatography
LCMS: Liquid chromatography mass spectrometry
L-DAP: L-2,3-diaminopropionic acid
NMR: Nuclear magnetic resonance
RAU: Relative absorbance unit
RO-Water: Reverse osmosis purified water
SIL: Stable-isotope-labelled
TMS-diazomethane: (trimethylsilyl)diazomethane
β-L-ODAP: β-L-oxalyl-2,3-diaminopropionic acid
